# The complete mitochondrial genome of *Monopis longella* Walker, 1863 (Lepidoptera: Tineidae)

**DOI:** 10.1080/23802359.2021.1944389

**Published:** 2021-07-01

**Authors:** Su Yeon Jeong, Jeong Sun Park, Min Jee Kim, Sung- Soo Kim, Iksoo Kim

**Affiliations:** a Department of Applied Biology, College of Agriculture & Life Sciences, Chonnam National University, Gwangju, Republic of Korea; b Experiment and Analysis Division, Honam Regional Office, Animal and Plant Quarantine Agency, Gunsan, Republic of Korea; c Research Institute for East Asian Environment and Biology, Republic of Korea

**Keywords:** Mitochondrial genome, *Monopis longella*, phylogeny, Tineidae

## Abstract

The complete mitochondrial genome (mitogenome) of *Monopis longella* Walker, 1863 (Lepidoptera: Tineidae) comprises 15,541 bp and contains a typical set of genes and one non-coding region. The gene arrangement of *M. longella* is unique for Lepidoptera in that it has a *trnI*-*trnM*-*trnQ* sequence in the A + T-rich region and *ND2* junction. Unlike most other lepidopteran insects, in which the *COI* gene has CGA as the start codon, *M. longella COI* has an ATT codon. Phylogenetic analyses based on the concatenated sequences of 13 protein-coding genes and two rRNA genes, using the Bayesian inference (BI) method, placed *M. longella* in the Tineidae, sister in position to the cofamilial species, *Tineola bisselliella*, with the highest nodal support. Tineidae, represented by three species including *M. longella*, formed a monophyletic group with high support (Bayesian posterior probability = 0.99). Within Tineoidea the sister relationship between Tineidae and Meessiidae was obtained with the highest support, leaving Psychidae occupying the basal lineage of the two families.

*Monopis longella* Walker, 1863 (Lepidoptera: Tineidae) is distributed in Korea, Japan, China, Russia (Far East), Thailand, Malaysia, The Philippines, Vietnam, Pakistan, and India (Huang et al. 2011; Lee et al. 2016). This species was initially recorded from Korea as *Monopis pavlovskii* Zagulajev, 1955 (Ponomarenko and Park 1996), but was later synonymized to *M. longella* based on morphological and mitochondrial *COI* data (Huang et al. 2011). The larvae of this moth feed on animal hair and feathers, typically living in tubular larval tunnels in the nests of birds (Huang et al. 2011).

In 2013, an adult male *M. longella* was collected from Jeollanam-do Province, South Korea (34°29′31.6′′ N, 126°16′07.0′′ E) and subsequently deposited as a voucher specimen at the Chonnam National University, Gwangju, Korea, under accession no. CNU7297 (Iksoo Kim, ikkim81@chonnam.ac.kr). DNA was extracted from the hind legs of this specimen using a commercial kit (Promega, Madison, WI, USA). Using this DNA three long overlapping fragments (LFs; *COI*-*ND4*, *ND5*-*lrRNA*, and *lrRNA*-*COI*) were amplified and served as templates for the amplification of 26 short overlapping fragments using the primers reported in Kim et al. (2012).

Phylogenetic analysis was performed using available species in the superfamilies Gracillarioidea, Yponomeutoidea, and Tineoidea (21 species including *M. longella*). Thirteen protein-coding genes (PCGs) and two rRNA genes including those of two outgroup species, were aligned using RevTrans ver. 2.0 (Wernersson and Pedersen 2003) and concatenated using SequenceMatrix ver. 1.8 (Vaidya et al. 2011). An optimal partitioning scheme (six partitions) and substitution model (GTR + Gamma + I) were determined using PartitionFinder 2 and the Greedy algorithm (Lanfear et al. 2012, 2014, 2016). Bayesian inference (BI) analysis that is implemented on the CIPRES Portal ver. 3.1 (Miller et al. 2010) were used for the phylogenetic analyses.

The complete 15,541 bp mitogenome of *M. longella* is composed of typical gene sets (two rRNAs, 22 tRNAs, and 13 PCGs) and a major non-coding A + T-rich region (GenBank acc. no. MH992770). The gene arrangement of the *M. longella* mitogenome is, however, unique in Lepidoptera, in that it has a *trnI*-*trnM*-*trnQ* (where underlining indicates a gene inversion) gene arrangement in the A + T-rich region and *ND2* junction. Previously, ditrysian Lepidoptera were reported to have the gene order *trnM*-*trnI*-*trnQ* at the same junction (Kim et al. 2010), which contrasts with the ancestral *trnI*-*trnQ*-*trnM* order found in the majority of insects (Boore 1999) including a few species of Lepidoptera (Cao et al. 2012; Wang et al. 2014). Twelve of the identified PCGs, including *COI*, contain a typical ATN start codon, whereas *ND5* has an infrequent TTG codon. The ATT start codon for *COI* differs from majority of other available species of Tineoidea, Gracillarioidea, and Yponomeutoidea (data not shown), as well as most species of Lepidoptera, which has CGA (Kim et al. 2012, 2018, 2020).

Phylogenetic analyses placed *M. longella* in the Tineidae in a sister position to the cofamilial species *Tineola bisselliella* with full support (Figure 1). Tineidae, represented by three species including *M. longella* formed a monophyletic group, with high nodal support (Bayesian posterior probability = 0.99) (Figure 1). Within the Tineoidea the sister relationship between the Tineidae and Meessiidae was also obtained with full support, leaving the Psychidae as the basal lineage of the two families. All families and superfamilies represented by multiple taxa formed respective monophyletic groups, with high nodal supports (Bayesian posterior probability = 0.99 − 1.0). Superfamilies in the Ditrysia showed the sister relationships between the Gracillarioidea and Yponomeutoidea, leaving Tineoidea as the basal lineage of the two superfamilies with the high supports (Figure 1). Currently, only limited species belonging to the Gracillarioidea, Yponomeutoidea, and Tineoidea are available for their mitogenome sequences in Ditrysia. In order to gain a more comprehensive picture of the phylogenetic relationships among lepidopteran superfamilies in Ditrysia, further analyses based on extended taxonomic sampling will be necessary.

**Figure 1.**
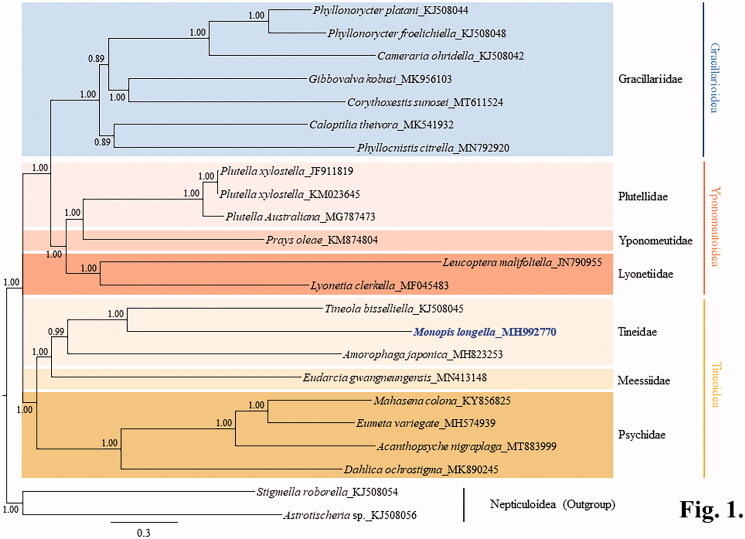
A Bayesian inference-based phylogenetic tree for three superfamilies in Ditrysia (Tineoidea, Gracillarioidea, and Yponomeutoidea) obtained using concatenated sequences of 13 protein-coding genes and two rRNAs. The numbers at each node indicate Bayesian posterior probabilities. The scale bar indicates the number of substitutions per site. Two species of Nepticuloidea (*Astrotischeria* sp. and *Stigmella roborella*) were included as an outgroup. GenBank accession numbers of the species analyzed are as follows: *Amorophaga japonica*, MH823253 (Kim et al. 2020); *Tineola bisselliella*, KJ508045 (Timmermans et al. 2014); *Eumeta variegata*, MH574939 (Jeong et al. 2018); *Mahasena colona*, KY856825 (Li et al. 2017); *Dahlica ochrostigma*, MK890245 (Roh et al. 2019); *Acanthopsyche nigraplaga*, MT883999 (Lee et al. 2021); *Eudarcia gwangneungensis*, MN413148 (Roh et al. 2020); *Phyllonorycter froelichiella*, KJ508048 (Timmermans et al. 2014); *Phyllonorycter platani*, KJ508044 (Timmermans et al. 2014); *Cameraria ohridella*, KJ508042 (Timmermans et al. 2014); *Caloptilia theivora*, MK541932 (Chen, Jiang, et al. 2019); *Gibbovalva kobusi*, MK956103 (Chen, Liao, et al. 2019); *Phyllocnistis citrella*, MN792920 (Liu et al. 2020); *Corythoxestis sunosei*, MT611524 (Zhang et al. 2020); *Plutella xylostella*, JF911819 and KM023645 (Wei et al. 2013; Dai et al. 2016); *Plutella australiana*, MG787473 (Wardz and Baxter 2018); *Leucoptera malifoliella*, JN790955 (Wu et al. 2012); *Lyonetia clerkella*, MF045483 (Unpublished); *Prays oleae*, KM874804 (van Asch et al. 2016); *Astrotischeria* sp., KJ508056 (Timmermans et al. 2014); *Stigmella roborella*, KJ508054 (Timmermans et al. 2014).

## Data Availability

The genome sequence data that support the findings of this study are openly available in GenBank of NCBI at https://www.ncbi.nlm.nih.gov/nuccore/MH992770.1.

## References

[CIT0001] Boore JL. 1999. Animal mitochondrial genomes. Nucleic Acids Res. 27(8):1767–1780. 10101183 10.1093/nar/27.8.1767 PMC148383

[CIT0002] Cao YQ, Ma C, Chen JY, Yang DR. 2012. The complete mitochondrial genomes of two ghost moths, *Thitarodes renzhiensis* and *Thitarodes yunnanensis*: the ancestral gene arrangement in Lepidoptera. BMC Genomics. 13:276. 22726496 10.1186/1471-2164-13-276 PMC3463433

[CIT0003] Chen L, Liao CQ, Wang X, Tang SX. 2019. The complete mitochondrial genome of *Gibbovalva kobusi* (Lepidoptera: Gracillariidae). Mitochondrial DNA Part B. 4(2):2769–2770. 33365720 10.1080/23802359.2019.1644550 PMC7706821

[CIT0004] Chen SC, Jiang HY, Shang J, Hu X, Peng P, Wang XQ. 2019. Characterization of the complete mitochondrial genome of the tea leaf roller *Caloptilia theivora* (Insecta: Lepidoptera: Gracillariidae). Mitochondrial DNA Part B. 4(2):2211–2212. 33365478 10.1080/23802359.2019.1624647 PMC7687574

[CIT0005] Dai LS, Zhu BJ, Qian C, Zhang CF, Li J, Wang L, Wei GQ, Liu CL. 2016. The complete mitochondrial genome of the diamondback moth, *Plutella xylostella* (Lepidoptera: Plutellidae). Mitochondrial DNA Part A. 27:512–513. 10.3109/19401736.2014.953116 25187437

[CIT0006] Huang GH, Chen LS, Hirowatari T, Nasu Y, Wang M. 2011. A revision of the *Monopis monachella* species complex (Lepidoptera: Tineidae) from China. Zool J Linn Soc. 163(1):1–14.

[CIT0007] Jeong JS, Kim MJ, Kim SS, Kim I. 2018. Complete mitochondrial genome of the female-wingless bagworm moth, *Eumeta variegata* Snellen, 1879 (Lepidoptera: Psychidae). Mitochondrial DNA Part B. 3(2):1037–1039. 33474406 10.1080/23802359.2018.1511851 PMC7799688

[CIT0008] Kim JS, Kim MJ, Jeong JS, Kim I. 2018. Complete mitochondrial genome of *Saturnia jonasii* (Lepidoptera: Saturniidae): genomic comparisons and phylogenetic inference among Bombycoidea. Genomics. 110(5):274–282. 29191682 10.1016/j.ygeno.2017.11.004

[CIT0009] Kim JS, Kim MJ, Kim SS, Kim I. 2020. Complete mitochondrial genome of *Amorophaga japonica* Robinson, 1986 (Lepidoptera: Tineidae). Mitochondrial DNA Part B. 5(3):2342–2344. 33457784 10.1080/23802359.2020.1774437 PMC7782908

[CIT0010] Kim JS, Park JS, Kim MJ, Kang PD, Kim SG, Jin BR, Han YS, Kim I. 2012. Complete nucleotide sequence and organization of the mitochondrial genome of eri-silkworm, *Samia cynthia ricini* (Lepidoptera: Saturniidae). J Asia Pac Entomol. 15(1):162–173.

[CIT0011] Kim MJ, Wan X, Kim KG, Hwang JS, Kim I. 2010. Complete nucleotide sequence and organization of the mitogenome of endangered *Eumenis autonoe* (Lepidoptera: Nymphalidae). Afr J Biotechnol. 9:735–754.

[CIT0012] Lanfear R, Calcott B, Ho SY, Guindon S. 2012. PartitionFinder: combined selection of partitioning schemes and substitution models for phylogenetic analyses. Mol Biol Evol. 29:1695–1701. 22319168 10.1093/molbev/mss020

[CIT0013] Lanfear R, Calcott B, Kainer D, Mayer C, Stamatakis A. 2014. Selecting optimal partitioning schemes for phylogenomic datasets. BMC Evol Biol. 14:82. 24742000 10.1186/1471-2148-14-82 PMC4012149

[CIT0014] Lanfear R, Frandsen PB, Wright AM, Senfeld T, Calcott B. 2016. PartitionFinder 2: new methods for selecting partitioned models of evolution for molecular and morphological phylogenetic analyses. Mol Biol Evol. 34:772–773. 10.1093/molbev/msw260 28013191

[CIT0015] Lee D-J, Ju Y-D, Bayarsaikhan U, Park B-S, Na S-M, Kim J-W, Lee B-W, Bae Y-S. 2016. First report on two species of genus *Monopis* (Lepidoptera, Tineidae) collected by feather trap in Korea. J Asia Pac Biodivers. 9(2):215–218.

[CIT0016] Lee KH, Kim MJ, Wang AR, Park JS, Kim SS, Kim I. 2021. Complete mitochondrial genome of *Acanthopsyche nigraplaga* (Lepidoptera: Psychidae). Mitochondrial DNA Part B. 6:091–1093. 10.1080/23802359.2021.1899876 PMC7995843 33796751

[CIT0017] Li PW, Chen SC, Xu YM, Wang XQ, Hu X, Peng P. 2017. The complete mitochondrial genome of a tea bagworm, *Mahasena colona* (Lepidoptera: Psychidae). Mitochondrial DNA Part B. 2(2):381–382. 33473834 10.1080/23802359.2017.1347839 PMC7800043

[CIT0018] Liu HL, Chen ZT, Chen S, Chen QD, Pu DQ, Liu YY, Liu X. 2020. Mitogenomic features of the citrus leafminer, *Phyllocnistis citrella* (Lepidoptera: Gracillariidae) and the related mitogenomic phylogeny. Mitochondrial DNA Part B. 5(3):2794–2795. 33457951 10.1080/23802359.2020.1787897 PMC7783059

[CIT0019] Miller MA, Pfeiffer W, Schwartz T. 2010. Creating the CIPRES Science Gateway for inference of large phylogenetic trees. Proceedings of the 9th Gateway Computing Environments Workshop (GCE), IEEE, 14 November 2010, New Orleans, LA; p. 1–8.

[CIT0020] Ponomarenko MG, Park KT. 1996. Notes on some Tineids from Korea and Russia Far East, with description of four new species (Lepidoptera: Tineidae). Kor J Appl Entomol. 35:273–279.

[CIT0021] Roh SJ, Kim DS, Lee BW, Byun BK. 2019. Complete mitochondrial genome of *Dahlica* (*Dahlica*) *ochrostigma* Roh and Byun, 2018 (Lepidoptera: Psychidae). Mitochondrial DNA Part B. 4(2):2922–2923. 33365793 10.1080/23802359.2019.1660243 PMC7706819

[CIT0022] Roh SJ, Kim IK, Byun BK. 2020. Complete mitochondrial genome of *Eudarcia gwangneungensis* (Lepidoptera: Meessiidae). Mitochondrial DNA Part B. 5(2):1746–1747.

[CIT0023] Timmermans MJ, Lees DC, Simonsen TJ. 2014. Towards a mitogenomic phylogeny of Lepidoptera. Mol Phylogenet Evol. 79:169–178. 24910155 10.1016/j.ympev.2014.05.031

[CIT0024] Vaidya G, Lohman DJ, Meier R. 2011. SequenceMatrix: concatenation software for the fast assembly of multi-gene datasets with character set and codon information. Cladistics. 27(2):171–180. 34875773 10.1111/j.1096-0031.2010.00329.x

[CIT0025] van Asch B, Blibech I, Pereira-Castro I, Rei FT, da Costa LT. 2016. The mitochondrial genome of *Prays oleae* (Insecta: Lepidoptera: Praydidae). Mitochondrial DNA Part A. 27:2108–2109. 10.3109/19401736.2014.982579 25423526

[CIT0026] Wang AR, Jeong HC, Han YS, Kim I. 2014. The complete mitochondrial genome of the mountainous duskywing, *Erynnis montanus* (Lepidoptera: Hesperiidae): a new gene arrangement in Lepidoptera. Mitochondrial DNA. 25(2):93–94. 23586336 10.3109/19401736.2013.784752

[CIT0027] Wardz CM, Baxter SW. 2018. Assessing genomic admixture between cryptic *Plutella* moth species following secondary contact. Genome Biol Evol. 10:2973–2985. 30321345 10.1093/gbe/evy224 PMC6250210

[CIT0028] Wei SJ, Shi BC, Gong YJ, Li Q, Chen XX. 2013. Characterization of the mitochondrial genome of the diamondback moth *Plutella xylostella* (Lepidoptera: Plutellidae) and phylogenetic analysis of advanced moths and butterflies. DNA Cell Biol. 32(4):173–187. 23496766 10.1089/dna.2012.1942

[CIT0029] Wernersson R, Pedersen AG. 2003. RevTrans: multiple alignment of coding DNA from aligned amino acid sequences. Nucleic Acids Res. 31(13):3537–3539. 12824361 10.1093/nar/gkg609 PMC169015

[CIT0030] Wu YP, Zhao JL, Su TJ, Li J, Yu F, Chesters D, Fan RJ, Chen MC, Wu CS, Zhu CD. 2012. The complete mitochondrial genome of *Leucoptera malifoliella* Costa (Lepidoptera: Lyonetiidae). DNA Cell Biol. 31(10):1508–1522. 22856872 10.1089/dna.2012.1642 PMC3458623

[CIT0031] Zhang ZT, Li J, Jin DC. 2020. The complete mitochondrial genome of *Corythoxestis sunosei* (Lepidoptera: Gracillariidae) with phylogenetic consideration. Mitochondrial DNA Part B. 5(3):2853–2854. 33457975 10.1080/23802359.2020.1790326 PMC7782291

